# iNOS Expression by Tumor-Infiltrating Lymphocytes, PD-L1 and Prognosis in Non-Small-Cell Lung Cancer

**DOI:** 10.3390/cancers12113276

**Published:** 2020-11-05

**Authors:** Alexandra Giatromanolaki, Avgi Tsolou, Eleftheria Daridou, Maria Kouroupi, Katerina Chlichlia, Michael I. Koukourakis

**Affiliations:** 1 Department of Pathology, Medical School, Democritus University of Thrace, 68100 Alexandroupolis, Greece; agiatrom@med.duth.gr (A.G.); marykouroupi@gmail.com (M.K.); 2 Department of Radiotherapy/Oncology, Medical School, Democritus University of Thrace, 68100 Alexandroupolis, Greece; atsolou@yahoo.gr (A.T.); patholdept@gmail.com (E.D.); 3 Department of Molecular Biology and Genetics, Democritus University of Thrace, University Campus Dragana, 68100 Alexandroupolis, Greece; achlichl@mbg.duth.gr

**Keywords:** lung cancer, iNOS, immune response, PD-L1, HIF1α

## Abstract

**Simple Summary:**

The role of Inducible Nitric Oxygen Synthase (iNOS) in the progression of human malignancies is obscure. We studied the expression patterns of iNOS in non-small-cell lung cancer. iNOS was expressed by cancer cells and cancer-associated fibroblasts. None of these patterns, however, are related to stage or prognosis. Extensive infiltration of the tumor stroma by iNOS-expressing tumor-infiltrating lymphocytes (^iNOS+^TILs) occurred in 48% of cases. This was related to low Hypoxia-Inducible Factor 1α (HIF1α) and better overall survival. Expression of Programmed death-ligand 1 PD-L1, however, mitigates the beneficial effect of the presence of ^iNOS+^TIL. An important role of iNOS in anti-neoplastic lymphocyte biology has been brought forward, supporting ^iNOS+^TILs as putative immune response markers.

**Abstract:**

Background: Inducible Nitric Oxygen Synthase (iNOS) promotes the generation of NO in tissues. Its role in tumor progression and immune response is unclear. Methods: The immunohistochemical expression patterns of iNOS were studied in a series of 98 tissue samples of non-small-cell lung carcinoma (NSCLC), in parallel with the expression of hypoxia and anaerobic metabolism markers, PD-L1 and tumor-infiltrating lymphocytes (TILs). Results: iNOS is expressed by cancer cells in 19/98 (19.4%), while extensive expression by cancer-associated fibroblasts occurs in 8/98 (8.2%) cases. None of these patterns relate to stage or prognosis. Extensive infiltration of the tumor stroma by iNOS-expressing TILs (^iNOS+^TILs) occurs in 47/98 (48%) cases. This is related to low Hypoxia-Inducible Factor 1α (HIF1α), high PD-L1 expression and a better overall survival (*p* = 0.002). Expression of PD-L1, however, mitigates the beneficial effect of the presence of ^iNOS+^TIL. Conclusions: Extensive expression of iNOS by TILs occurs in approximately 50% of NSCLCs, and this is significantly related to an improved overall survival. This brings forward the role of iNOS in anti-neoplastic lymphocyte biology, supporting ^iNOS+^TILs as a putative marker of immune response. The value of this biomarker as a predictive and treatment-guiding tool for tumor immunotherapy demands further investigation.

## 1. Introduction

Nitric Oxygen Synthases (NOS) catalyze the conversion of arginine to citrulline, a reaction that produces nitric oxide (NO). Constitutive NO synthases (neuronal and endothelial NOS) produce NO at low quantities, while Inducible Nitric Oxygen Synthase (iNOS) generates large amounts of NO at short intervals [1]. The expression levels of iNOS go along with the quantity of NO generated by cells and tissues [2]. NO seems to have a dual role in tumorigenesis and tumor progression. The NO oxidative pathway induces S-nitrosylation of various proteins, including Hypoxia-Inducible Factor 1α (HIF1α), Nuclear Factor κΒ(NFκB), matrix metalloproteinases or caspases, activating death or pro-survival pathways, depending upon the cell system and microenvironmental conditions [3].

Experimental data suggest that the NO concentration is critical in order to define the nature of NO effects. Low concentrations, below 100nM, favor ERK phosphorylation, promoting cell survival, proliferation and apoptosis inhibition [4]. High concentrations, above 500nM, are cytotoxic, promote p53 phosphorylation and have anti-tumor anti-metastatic effects [5,6]. Immune cells producing high amounts of NO, like macrophages or even lymphocytes, have strong anti-tumor activities [7,8]. NO reacts with superoxide to form the cytotoxic molecule peroxynitrite [9], which has a potent anti-microbial and tumoricidal activity. The role of NO produced by tumor stroma fibroblasts or cancer cells themselves in cancer progression remains controversial [10,11].

We therefore studied the expression of iNOS, in cancer cells, tumor stroma and tumor-infiltrating lymphocytes (TILs), in a series of non-small-cell lung carcinomas (NSCLC). iNOS correlation with markers of intratumoral hypoxia and acidity, and markers of immune response, was also investigated. In addition, the impact of iNOS expression on the survival of patients was examined.

## 2. Results

### 2.1. Expression of iNOS in Normal Lung

**Figure 1:**
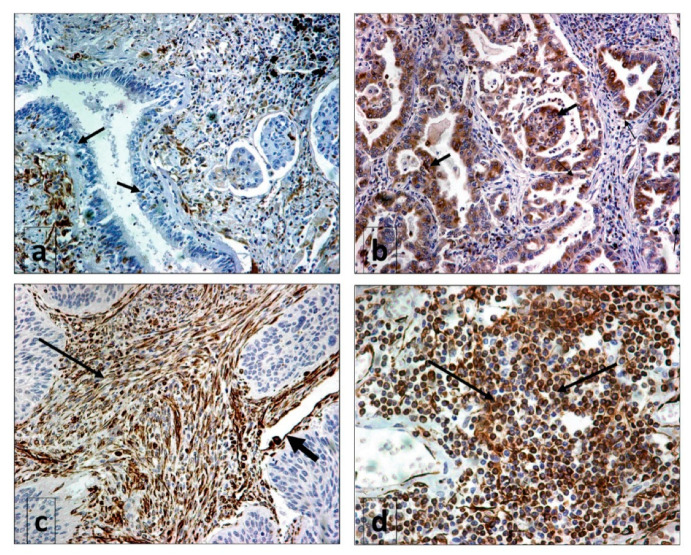
Typical immunohistochemical images of Inducible Nitric Oxygen Synthase (iNOS) expression: (**a**) lack of expression in bronchial epithelium (arrows) (magnification ×20); (**b**) strong cytoplasmic expression by cancer cells (arrows) (magnification ×20); (**c**) strong expression by stroma fibroblasts (thin arrow) and a tumor vessel (thick arrow) in the context of negative cancer cell expression (magnification ×40); (**d**) extensive infiltration of the tumor stroma by iNOS-expressing lymphocytes (arrows) (magnification ×40).

The bronchial and alveolar epithelium, as well as the glandular epithelium, displayed a very weak (considered negative) expression (Figure 1a). Alveolar macrophages were strongly positive. Normal vessels sporadically expressed iNOS.

### 2.2. Expression of iNOS in Cancer

iNOS was expressed by both cancer cells and tumor stroma fibroblasts (Cancer-Associated Fibroblasts—CAFs) to a varying extent (Figure 1b,c). Tumor vessels were occasionally positive (Figure 1c). Infiltrating lymphocytes extensively expressed iNOS in a large fraction of tumor samples (Figure 1d).

In 55 (56.1%) cases, out of 98 examined, cancer cells were negative. iNOS expression by cancer cells was noted in 43 cases (43.9%), ranging from 1 to 80% (median 5%). In 24/98 (24.5%) cases, expression of iNOS was noted in less than 10% of cancer cells and these were considered to have low reactivity, while medium/high reactivity (expression in 10–80 of cancer cells) was noted in 19/98 (19.4%) cases (Table 1).

Analysis of the extent of expression in the tumor stroma CAFs showed that a lack of iNOS expression occurred in 83/98 cases (negative 84.7%), while expression in 5–90% of the area of the stroma was noted in 15/98 (15.3%) cases. Out of these 15 cases, seven had low expression (focal expression occupying <10% of the stroma area), while eight had medium/high expression (10–100% of the stroma) (Table 1).

Analysis of the stroma infiltration by iNOS+ lymphocytes showed that, in 42/98 (42.8%) of cases, no lymphocytes were stained for iNOS (negative). In the remaining 56 cases, the percentage of lymphocytes stained with iNOS ranged from 2 to 90% (median 10%). In 33/98 (33.7%) cases, this percentage ranged from 2 to 9% (low) and in 23/98 (23.5%) from 10 to 90% (medium/high) (Table 1). Studying the spatial distribution of ^iNOS+^TILs in tumors with high iNOS+ lymphocyte expression showed that inner tumor areas had a higher ^iNOS+^TIL density compared to the tumor invasion front (median 20% vs. 8%; *p* = 0.0001). In order to examine whether iNOS preferentially stained T- or B-lymphocytes, we performed double immunostaining for CD4/iNOS, CD8/iNOS and CD20/iNOS in 10 selected tissue samples with the intense presence of ^iNOS+^TILs. All three lymphocytic subpopulations showed the expression of iNOS in 10–30% of the total CD4+, CD8+ or CD20+ TILs.

The ^iNOS+^TIL score (see Methods) ranged from 0 to 3.6 (median 0.1). In 42/98 (42.9%), this score was zero (score 0). In 9/98 (9.2%), it was 0.04–0.1 (score 1). In 23/98 (23.5%), it ranged between 0.15 and 0.3 (score 2) and, in the remaining 24/98 (24.5%), between 0.4 and 3.6 (score 3) (Table 1).

### 2.3. Association of iNOS Expression with Histopathological Parameters

Analysis of iNOS expression according to the histology showed a significant increased expression in cancer cells and in Cancer-Associated Fibroblasts in tumors with squamous histology (*p* = 0.02 and 0.01, respectively, Table 2). The ^iNOS+^TIL score was also significantly related to squamous cell histology (*p* = 0.03). No association with the stage of disease was detected (Table 2).

### 2.4. Correlation of iNOS Expression with Hypoxic Markers

**Figure 2:**
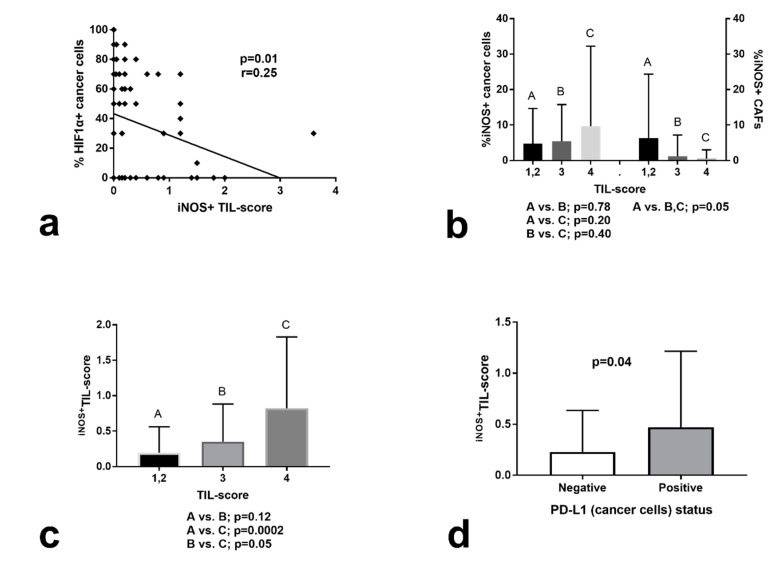
Statistical associations of iNOS expression: (**a**) Linear regression analysis of ^iNOS+^TIL score vs. the extent of Hypoxia-Inducible Factor 1α (HIF1α) expression by cancer cells. (**b**) Percentage of iNOS+ cancer cells and extent of iNOS expression by Cancer-Associated Fibroblasts (CAFs) according to the TIL score. (**c**) Association between ^iNOS+^TIL score and TIL score. (**d**) ^iNOS+^TIL score in tumors with positive and negative PD-L1 cancer cell expression.

iNOS expression in cancer cells or in CAFs was not associated with the expression of HIF1α, isoenzymes of lactate dehydrogenase, involved in the anaerobic transformation of pyruvate to lactate (LDH5) or CA9 by cancer cells. Interestingly, although HIF1α expression did not relate to TIL score, this was inversely related to the ^iNOS+^TIL score (*p* = 0.01, *r* = 0.25; Figure 2a). LDH5 expression was inversely related to TIL score (*p* = 0.04, *r* = 0.20), but no association was noted with ^iNOS+^TIL score. CA9 was not related to either TIL or ^iNOS+^TIL scores.

### 2.5. Correlation of iNOS Expression with Immunological Parameters

iNOS expression by cancer cells was not linked to TIL score. On the contrary, iNOS expression by Cancer-Associated Fibroblasts (CAFs) was inversely linked to TIL score (*p* = 0.05; Figure 2b). ^iNOS+^TIL score was directly linked to TIL score (*p* = 0.0002) (Figure 2c). PD-L1 expression by cancer cells was not related to iNOS expression by either cancer cells or CAFs. A lack of expression of PD-L1, however, was related to a low ^iNOS+^TIL score (*p* = 0.04; Figure 2d).

### 2.6. Survival Analysis

**Figure 3:**
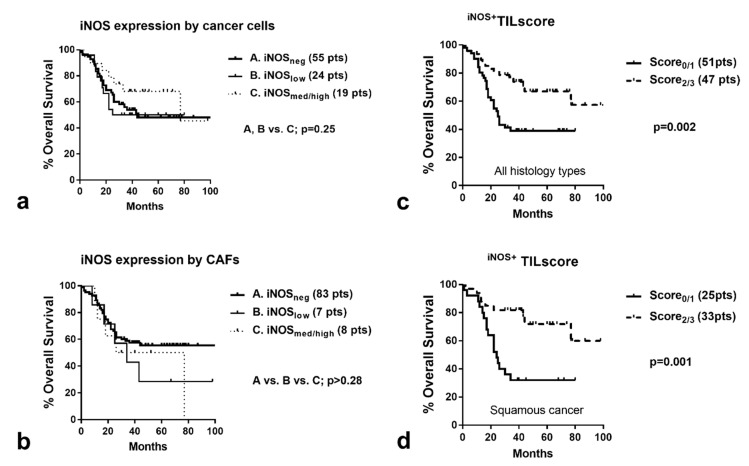
Kaplan–Meier (disease-specific) overall survival curves stratified for (**a**) iNOS expression by cancer cells; (**b**) iNOS expression by Cancer-Associated Fibroblasts; (**c**) ^iNOS+^TIL score in all histology types; (**d**) ^iNOS+^TIL score in squamous cell histology.

There was no association of iNOS expression in cancer cells or CAFs with overall survival (Figure 3a,b). A high ^iNOS+^TIL score was significantly linked to a better prognosis (*p* = 0.002) (Figure 3c). Stratification according to ^iNOS+^TIL score in the three histology subtypes showed significant associations with prognosis in squamous cell carcinomas (*p* = 0.001; Figure 3d) but not in adenocarcinomas (*p* = 0.86). In large-cell cancer, the difference was marginal due to the small number of cases (*p* = 0.10). In a bivariate model, stage (*p* < 0.0001, HR 1.9 (95%, with Confidence Interval (CI) 1.3–2.6)) and ^iNOS+^TIL score (*p* = 0.001. HR = 0.3 (95% CI 0.1–0.7)) were independent prognostic variables.

**Figure 4:**
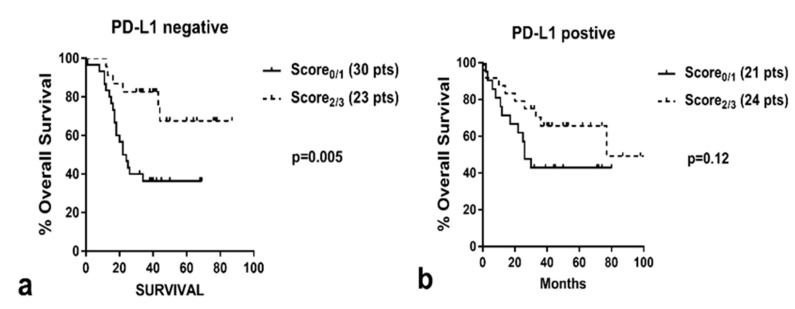
Kaplan–Meier (disease-specific) overall survival curves stratified for ^iNOS+^TIL score (**a**) in tumors with lack of PD-L1 expression in cancer cells, and (**b**) in tumors positive for PD-L1 expression in cancer cells.

As ^iNOS+^TIL score was directly linked to PD-L1 expression, a bivariate analysis of survival was performed (Figure 4) to assess the prognostic role of this TIL feature on the prognosis of PD-L1-positive and -negative tumors. Intense stroma infiltration by ^iNOS+^TILs was significantly related to a better prognosis in patients with PD-L1-negative tumors (*p* = 0.005). In PD-L1-positive tumors, however, ^iNOS+^TIL score had a less potent prognostic impact, with the difference not reaching significance (*p* = 0.12).

## 3. Discussion

The role of iNOS in the development and progression of cancer remains unclear and is under intense investigation. Its prognostic role in human malignancies is also under examination. A main factor that hampers the attempts to reach reliable conclusions is the fact that iNOS is expressed not only in cancer cells, but also in stroma fibroblasts and tumor-infiltrating immune cells. Therefore, each expression pattern may have distinct, or even opposing, relevance to the tumor biology and clinical behavior of tumors. A recent meta-analysis suggested that iNOS expression has an ominous prognostic impact in solid tumors, but the analysis was based on immunohistochemical data provided by published studies that used different cut-off points of iNOS expression and, furthermore, the analysis did not focus on the distinct patterns of expression within tumors [12]. For example, overexpression of iNOS by tumor-associated macrophages has been linked to a favorable prognosis in breast and lung cancer [13,14]. Moreover, several studies demonstrate that expression of iNOS by cancer cells is linked to a better prognosis. For example, iNOS expression has been linked to high apoptotic rates and reduced post-therapy recurrence of the disease in patients with nasopharyngeal tumors [15]. Similarly, low iNOS expression has been linked to tumor recurrence and poor survival in ovarian cancer [16]. To the best of our knowledge, there are no clinicopathological studies focusing on the distinct iNOS expression patterns in tumors and how these relate to tumor growth, metastasis or prognosis. Such an (as yet) absent analysis would contribute to the clarification of the complex role of iNOS in human tumors.

In the current study, we focused on the expression of iNOS in NSCLC tissues. In normal lung tissues, iNOS was not expressed in the bronchial epithelial cells, although lung macrophages displayed an intense expression. In tumor tissues, iNOS was expressed in three distinct compartments—cancer cells, cancer-associated fibroblasts (CAFs) and tumor-infiltrating lymphocytes (TILs). Regarding cancer cells, a lack of iNOS expression was noted in more than half of tissues examined, while extensive expression in more than 10% of cancer cells was noted in only 20% of tumors. The latter pattern was significantly more frequent in tumors of squamous histology but there was no association with local tumor stage or metastasis to the lymph nodes. A trend for a better prognosis in patients with high cancer cell iNOS expression was noted, but this did not reach significance. The published data on the prognostic role of iNOS in NSCLC are limited. A study by Puhakka et al. reported a significant association of iNOS expression with better prognosis in NSCLC [17]. In contrast, Rubio et al. found an ominous prognostic impact of iNOS expression [18].

Regarding the expression of iNOS by the tumor stroma fibroblasts (or CAFs), our study showed that most tumors (85%) were negative, suggesting that iNOS of CAF origin has a limited role in a small fraction of NSCLCs. Indeed, extensive expression of iNOS by CAFs was evident in less than 10% of tumors examined. This expression pattern concerned a subset of squamous cell carcinomas and had no apparent prognostic relevance. An interesting finding, however, was that tumors with extensive iNOS expression by CAFs were immunologically cold, with poor infiltration of TILs in their stroma.

In contrast to the scarcity of iNOS expression by CAFs, a high percentage of TILs had extensive expression of iNOS and a dense infiltration of the tumor stroma by ^iNOS+^TILs (high ^iNOS+^TIL score) was evident in about half of tumors. This pattern was, once again, more frequent in tumors of squamous histology and was not related to the stage of the disease. The ^iNOS+^TIL subpopulation reached up to 90% of the total TILs in some cases, suggesting that iNOS is expressed by more than one subtype of lymphocytes. This feature was strongly related to a better postoperative prognosis, showing that iNOS expression by TILs is a potent marker of activated cytotoxic immune activity, and thus an eventual marker of effector immune cells. Interestingly, a strong association of HIF1α expression by cancer cells with low ^iNOS+^TIL score was noted, bringing forward hypoxia as a micro-environmental condition that sustains a cold immune environment [19].

Not much is known about iNOS-expressing lymphocytes. A subset of activated T-cells produces NO, and iNOS could be involved in T-cell differentiation [20]. An interesting study by Scheller et al. showed that nitric oxide is essential for the activity of a parasite-specific subset of CD8 cells that were responsible for the clearance of malaria parasites from hepatocytes [21]. It is also known that iNOS is essential for the secretion of NO by tumor-infiltrating M1-type monocytes. This results in the production of peroxynitrite, which triggers apoptosis, a mechanism that could also be used by ^iNOS+^TILs. Infiltration of colon carcinomas by iNOS-positive macrophages has been related to a better prognosis [22], but there are no published data on the prognostic role of iNOS-expressing TILs in lung cancer or other human malignancies. Interestingly, iNOS expression in CD4+ lymphocytes seems to suppress the induction of regulatory T-cells, suggesting that an abundance of iNOS-expressing TILs in tumors goes hand in hand with effective TIL activity [23].

The finding that PD-L1 expression by cancer cells is related to a significantly higher infiltration of NSCLCs with ^iNOS+^TILs deserves further investigation. In such cases, PD-L1 could block the tumoricidal activity of ^iNOS+^TILs. Indeed, the prognostic value of ^iNOS+^TIL score was lost in patients with PD-L1-positive tumors. If suppression of the anti-tumor activity of PD-L1 on ^iNOS+^TILs occurs, anti-PD-L1 therapies may be more effective in NSCLCs with PD-L1+/high ^iNOS+^TIL score tumors compared to PD-L1+ cold tumors. Whether lymphocytic iNOS can be used as a biomarker to enhance the predictive value of PD-L1 in NSCLC is a hypothesis that emerges from the current study.

## 4. Materials and Methods

### 4.1. Patient and Disease Characteristics

The study was performed on a series of 98 tissue samples of surgically resected non-small-cell lung cancer (NSCLC). All patients were treated with surgery alone. The median age of patients was 68 years, ranging from 32 to 82 years. The Union for International Cancer Control (UICC) staging showed that 46 patients were of stage I, 22 of stage II and 30 of stage III. Squamous histology was confirmed in 58 cases, while 22 cases were adenocarcinomas and 18 undifferentiated large-cell carcinomas. The median follow-up of patients was 46 months (range: 26–112 months).

### 4.2. Ethical Considerations

Ethical approval was obtained from the Scientific Committee and the Ethics Research Committee of the University Hospital of Alexandroupolis (study approval number ES11-26-11-18). The study was conducted according to the criteria set by the Declaration of Helsinki.

### 4.3. iNOS Immunohistochemistry

Formalin-fixed paraffin-embedded material was retrieved from the archives of the Department of Pathology, University Hospital of Alexandroupolis. Tissue sections 3 μm thick were placed on positively charged slides. For the detection of iNOS, we used the primary rabbit polyclonal ab15323 antibody (Abcam, Cambridge, UK), with overnight incubation, at a dilution of 1/50. In order to stain for iNOS, the slides were deparaffinized by xylene and rehydrated in graded ethanol solutions. The heat-induced epitope retrieval process was performed in a microwave oven using Dako EnVision FLEX Target Retrieval Solution (pH 9.0). After washing the specimen twice for 6 min each time, the slides were incubated with primary antibody. Endogenous peroxidase was quenched with EnVision Flex Peroxidase Block (DAKO, Glostrup, Denmark) for 10 min. For iNOS immunostaining, slides were subsequently incubated with the secondary antibody (EnVision Flex/HRP; DAKO) for 30 min, and washed in Phosphate Buffer Saline (PBS). The color was developed after 5 min incubation with EnVision Flex Chromogen (DAKO). Sections were counterstained with hematoxylin. In every staining run, a negative control section was included by replacing the primary antibody with normal rabbit immunoglobulin-G.

Assessment of the expression of iNOS was performed at ×200 magnification. The percentage of cancer cells with strong cytoplasmic expression was recorded in all optical fields and the mean value was calculated and used to score each tissue section. The extent of Cancer-Associated Fibroblasts (CAFs) stained with iNOS was recorded as the percentage of stained stromal area in the tissue section, at ×200 magnification. The percentage of tumor-infiltrating lymphocytes (TILs) stained for iNOS (^iNOS+^TILs within total TIL presence) was also recorded in all ×400 optical fields and the mean score was used to characterize each case.

### 4.4. Assessment of Other Immunohistochemical Markers

The expression of PD-L1 by cancer cells was assessed using immunohistochemistry with the rabbit monoclonal anti-PD-L1 antibody (clone CAL10, BioCare Medical, Pacheco, CA, USA), as previously reported [23]. The percentage of cancer cells with strong membrane (with or without cytoplasmic) expression was recorded in all ×200 o.f. (Optical field), and the mean value was used to score each case. Cases were grouped into three categories: negative (lack of reactivity); low (expression in 1–9% of the cancer cells); high (expression in >10% of the cancer cells). Low and high expression defined tumors positive for PD-L1.

The expression of isoenzymes of lactate dehydrogenase, involved in the anaerobic transformation of pyruvate to lactate (LDH5), Hypoxia-Inducible Factor (HIF1α; the transcription factor regulating metabolism under hypoxic conditions) and of carbonic anhydrase 9 (CA9; involved in the transformation of carbon dioxide to carbonic acid and the acidification of tumor stroma) were assessed using the ab9002 (Abcam, UK), the ESEE 122 (Oxford, UK) and the M75 antibodies (Oxford, UK), respectively, as previously described [24,25].

### 4.5. Scoring of TIL and ^iNOS+^TIL Density

Tumor-infiltrating lymphocytes were quantified on hematoxylin-stained tissue sections, as previously described [12]. Briefly, the number of TILs infiltrating the tumor stroma only (excluding tumor nests), was assessed in all ×40 optical fields. The mean value was obtained to score each case. These scores were subsequently grouped into four different groups, corresponding to 4 different TIL scores as follows: 1 (minimal, mean value 1–10 lymphocytes/o.f.), 2 (low, mean value 10–70 lymphocytes/o.f.), 3 (medium, mean value 70–150 lymphocytes/o.f.), and 4 (high, mean value >150 lymphocytes/o.f.).

As the percentage of iNOS+ lymphocytes shows only the relative presence of such lymphocytes in the total TIL population and does not take into account the overall TIL density (which can be sparse or dense), the extent of stroma infiltration by ^iNOS+^TILs was calculated by multiplying the % ^iNOS+^TILs with the TIL score (which ranges from 1 to 4; see Methods). In this way, we produced the ‘^iNOS+^TIL score’.

### 4.6. Statistical Analysis

The GraphPad Prism 7.0 package was used for statistical analysis and graph presentation. The chi-square test or Fisher’s exact *t*-test were applied to compare categorical variables, as appropriate. Pearson’s correlation analysis was used to assess relations between continuous variables. Kaplan–Meier survival curves were applied to assess the impact of immunohistochemical variables on the overall disease-specific survival. A Cox proportional hazard model was used for multivariate analysis of death events. A *p*-value of <0.05 was considered for significance.

## 5. Conclusions

It is concluded that iNOS is expressed in both cancer cells and fibroblasts, in a small subgroup of NSCLCs—mainly of squamous histology—which, however, does not seem to have any robust effect on lung cancer clinical behavior. The striking and new evidence provided by the current study is the extensive expression of iNOS by TILs in about half of tumors, which was significantly correlated with improved overall survival. This suggests a role of iNOS in anti-neoplastic lymphocyte biology, supporting lymphocyte iNOS as a putative biomarker of immune response that may prove to be of significance even as a predictive and treatment-guiding marker for tumor immunotherapy.

## Figures and Tables

**Table 1:** Expression patterns of iNOS in tumor tissues.

Extent of Cancer Cell expression		
Pattern	% cancer cell reactivity	*No* pts (%)
Negative	0	55 (56.1%)
Low	1–9	24 (24.5%)
Medium/High	10–100	19 (19.4%)
Extent of expression by Cancer-Associated Fibroblasts		
Pattern	% stroma reactivity	*No* pts (%)
Negative	0	83 (84.7)
Low	1–9	7 (7.1)
Medium/High	10–100	8 (8.2)
Percentage of ^iNOS+^TILs		
Pattern	% TILs	*No* pts (%)
Negative	0	42 (42.8%)
Low	1–9	33 (33.7%)
Medium/High	10–100	23 (23.5%)
^iNOS+^TIL score		
Pattern	% TILs	*No* pts (%)
0	0	42 (42.9%)
1	0.04–0.1	9 (9.2%)
2	0.15–0.3	23 (23.5%)
3	0.4–3.6	24 (24.5%)

**Table 2:** Association of iNOS expression with histopathological parameters.

**Histology**				
Cell Type	S	A	L	*p*-value
Cancer Cells				
Negative (55)	26	17	12	0.02 (Sq vs. other)
Low (24)	17	4	3	
Med/High (19)	15	1	3	
Stroma CAFs				
Neg (83)	45	21	17	0.01 (Sq vs. other)
Low (7)	6	0	1	
Medium/High (8)	7	1	0	
^iNOS+^TIL score				
0/1 (51)	25	14	12	0.03 (Sq vs. other)
2/3 (47)	33	8	6	
**Stage**				
Cell Type	1	2	3	*p*-value
Cancer Cells				
Negative (55)	27	10	18	0.61
Low (24)	12	7	5	
Med/High (19)	7	5	7	
Stroma CAFs				
Neg (83)	40	18	25	0.83
Low (7)	3	2	2	
Medium/High (8)	3	2	2	
^iNOS+^TIL score				
0/1 (51)	24	11	16	0.97
2/3 (47)	22	11	14	
